# Data-driven stochastic modelling of zebrafish locomotion

**DOI:** 10.1007/s00285-014-0843-2

**Published:** 2014-10-31

**Authors:** Adam Zienkiewicz, David A.W. Barton, Maurizio Porfiri, Mario di Bernardo

**Affiliations:** 1 Department of Engineering Mathematics, University of Bristol, Bristol, UK; 2 Department of Electrical Engineering and ICT, University of Naples Federico II, Naples, Italy; 3 Department of Mechanical and Aerospace Engineering, New York University Polytechnic School of Engineering, New York, USA

**Keywords:** Fish locomotion, Computational biology, Stochastic models, Zebrafish, Ornstein–Uhlenbeck, 37N25, 46N60, 97M60, 60H10, 91B70, 37B70

## Abstract

In this work, we develop a data-driven modelling framework to reproduce the locomotion of fish in a confined environment. Specifically, we highlight the primary characteristics of the motion of individual zebrafish *(Danio rerio)*, and study how these can be suitably encapsulated within a mathematical framework utilising a limited number of calibrated model parameters. Using data captured from individual zebrafish via automated visual tracking, we develop a model using stochastic differential equations and describe fish as a self propelled particle moving in a plane. Based on recent experimental evidence of the importance of speed regulation in social behaviour, we extend stochastic models of fish locomotion by introducing experimentally-derived processes describing dynamic speed regulation. Salient metrics are defined which are then used to calibrate key parameters of coupled stochastic differential equations, describing both speed and angular speed of *swimming* fish. The effects of external constraints are also included, based on experimentally observed responses. Understanding the spontaneous dynamics of zebrafish using a bottom-up, purely data-driven approach is expected to yield a modelling framework for quantitative investigation of individual behaviour in the presence of various external constraints or biological assays.

**Electronic supplementary material:**

The online version of this article (doi:10.1007/s00285-014-0843-2) contains supplementary material, which is available to authorized users.

## Introduction

The coordinated motion of groups of animals, and in particular of fish shoals, has been widely studied both from experimental and theoretical perspectives (Partridge 1982; Huth and Wissel 1992; Camazine et al. 2001; Couzin et al. 2002; Krause and Ruxton 2002; Buhl et al. 2006; Kolpas et al. 2007; Pillot et al. 2011; Moussaïd et al. 2011; Gautrais et al. 2012; Vicsek and Zafeiris 2012). Numerous authors have proposed a wide variety of theoretical models, for example the canonical Vicsek-type models (Vicsek et al. 1995) along with those by Czirók et al. (1997, 1999) which describe mobile agents as identical self-propelled particles with heading directions updated via the integration of noisy local-neighbourhood interaction rules. More elaborate models of collective motion have also been proposed which may account for repulsive and attractive forces between fish (or other animals), for example those by Aoki (1982), Reynolds (1987), Huth and Wissel (1992), Couzin et al. (2002), Grégoire and Chaté (2004), Chaté et al. (2008), Kolpas et al. (2013). Within all of these models, the dynamics of the group behaviour are dissected into individual rules from which complex coordinated motions emerge. It is critical therefore to establish predictive and tractable models for the behavioural response of isolated individuals, upon which to study and construct models of sociality.

The degree to which individual behaviour modulates group dynamics, and correspondingly, how interactions with conspecifics affects individual response, can be tested with a modelling cycle driven by precise experimental data. The recent work of Gautrais et al. serves as an important example of this process. Specifically, a data-driven model of spontaneous fish movement was first derived by Gautrais et al. (2009). Then, using a bottom-up methodology, a model of group motion from data gathered at the level of the individual was developed by Gautrais et al. (2012). Unlike many other models of collective motion, this approach enables all model parameters to be estimated directly from experimental data. Based on evidence that the fish considered (*Kulia Mugil*) are best described in terms of their turning speed and its autocorrelation, Gautrais et al. have developed a model referred to as a ‘Persistent Turning Walker’ (PTW). This model is based on an Ornstein–Uhlenbeck (O-U) stochastic differential equation (SDE) governing the turning speed of an agent with a fixed forward speed. In addition, the effects of environmental confinement were considered, providing a versatile methodology for incorporating fixed boundaries, obstacles and other fish, within the same model framework.

Recent experimental studies, including those by Krause and Ward (2005), Herbert-Read and Perna (2011), Katz et al. (2011), Berdahl et al. (2013), Herbert-Read et al. (2013), show that the speed response of fish play an important role in fish interaction. For example, a comprehensive study by Katz et al. (2011) reveals the subtle modulation of turning and speeding responses of groups of golden shiners in relation to their conspecifics. Important conclusions from this work include the observation that speed regulation may be a dominant component of interaction, where subsequent alignment between neighbouring fish emerges from the interplay between attraction and repulsion. With respect to this latter conclusion, some models including that of Strömbom (2011), have demonstrated the characteristic hallmarks of collective motion with a rich diversity of dynamics such as swarming, and circular and directed milling, emerging solely from inertial local attraction between individuals. The study by Katz et al. also examined the importance of higher order interactions, namely a non-trivial 3-body component which may contradict the pervasive assumption of models which exclusively integrate pairwise interactions. Supporting experimental work by Herbert-Read and Perna (2011) also suggested the absence of an empirically justifiable alignment rule for schooling mosquitofish, suggesting that group polarisation is an emergent property. In this study, speed regulation was again found to be a key reaction mechanism due to group interactions, especially repulsion from close neighbours, with clearly defined zones of interaction.

Most commonly, models of schooling consider fish as agents with fixed forward speed (Couzin et al. 2002; Gautrais et al. 2009, 2012), and thus prevent us from exploring the role of speed regulation in collective dynamics. Existing models of collective motion which do consider variable speed agents (Reynolds 1987; Huth and Wissel 1994; Toner and Tu 1998; D‘Orsogna et al. 2006; Strefler et al. 2008; Ebeling and Schimansky-Geier 2008; Abaid and Porfiri 2010; Strömbom 2011; Mishra et al. 2012) either describe self-propelled particles as a continuum dynamical system, or rather consider the effects of noise on the absolute velocity. Thus far however, none of these approaches have been fully validated against experimental data in terms of their description of speed modulation.

The primary aim of this paper is to extend the approach by Gautrais et al. (2009) to develop a data-driven modelling framework describing the individual locomotion of zebrafish. Selected here primarily for their strong propensity to form social groups (Miller and Gerlai 2008; Saverino and Gerlai 2008; Miller and Gerlai 2012), laboratory studies with zebrafish also benefit from their short intergenerational time and comparatively high reproductive rate. Yielding extensive genomic homologues with both humans and rodents, zebrafish have emerged as one of the predominant species for neurobiological, developmental and behavioural studies (Gerlai 2003; Kuo and Eliasmith 2005; Miklósi and Andrew 2006; Lawrence 2007; Kalueff et al. 2014). In this work, we find that modelling zebrafish motion requires an additional, experimentally calibrated process governing the variation of swimming *speed*. In light of recent studies such as those by Katz et al. (2011), Herbert-Read and Perna (2011), Berdahl et al. (2013), indicating that speed regulation is a key response of similar fish to external stimuli, this latter modification represents a shift away from many canonical models, which prescribe a constant speed, and provides the foundations for a novel modelling approach for studying zebrafish social behaviour.

The modelling process addressed in this work employs a bottom-up approach, using data analysed from experimentally observed zebrafish trajectories, primarily in terms of position/velocity time-series data, to inform an empirical model of individual swimming locomotion. Based on the direct analysis of experimental zebrafish trajectory data obtained via automated computer vision techniques at the Dynamical Systems Laboratory (New York University Polytechnic School of Engineering, NY, USA), we clarify whether the stochastic PTW models of spontaneous fish motion, developed by Gautrais et al. (2009), can be applied to suitably describe the locomotion of zebrafish. We wish to emphasise that the modelling framework presented in this paper can provide the foundation for future extensions which capture group level dynamics of zebrafish shoals and their interaction with semi-autonomous artificial stimuli (Aureli et al. 2012; Kopman et al. 2012; Aureli et al. 2010; Aureli and Porfiri 2010).

## Materials and methods

### Ethics statement

The experimental data for this analysis was provided by the Dynamic Systems Laboratory, New York University Polytechnic School of Engineering, NY, USA. Trajectory data for isolated fish analysed in this study are derived from source data published in the recent work of Butail et al. (2014) (‘No Robot’ control condition). All experiments were conducted following the protocols AWOC-2012-101 and AWOC-2013-103 approved by the Animal Welfare Oversight Committee of the New York University Polytechnic School of Engineering.

### Animals and environment

Wild-type zebrafish *(Danio rerio)* were used in all experiments, acquired from an online aquarium (LiveAquaria.com, Rhinelander, Wisconsin, USA). Subjects age was between 6 to 8 months, inferred from their average body length (BL) of approximately 3 cm. Fish were kept in 37.8 l (10 US gallon) holding tanks with a maximum of 20 individuals in each. A photoperiod of 12 h light / 12 h dark was sustained prior to experimentation as per Cahill (2002). Water temperature in the holding tanks was maintained at $27 \pm 1~^\circ$C with a pH of 7.2. Fish were fed daily at 7 pm with commercial flake food (Hagen Corp./Nutrafin Max, USA). Experiments were started after a 10 days acclimatization period.

### Apparatus

**Fig. 1:**
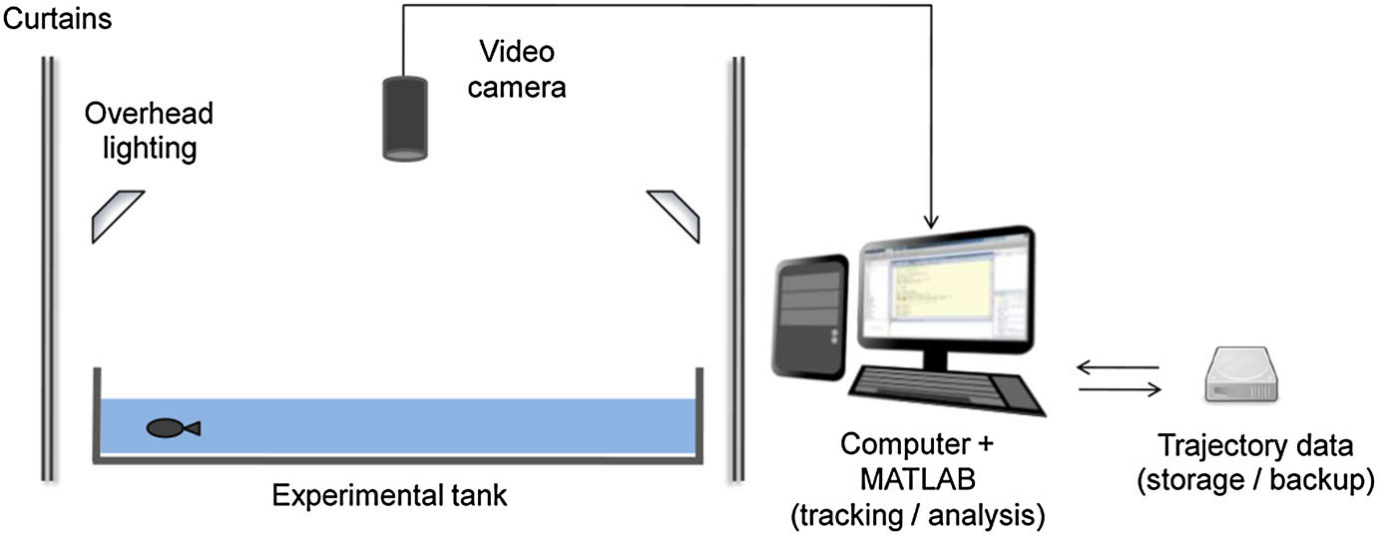
Schematic of experimental apparatus for observing, tracking and analysing free-swimming zebrafish. Fish are transferred to experimental tank ($120\times 120\times 20\,\hbox {cm})$ with water level of 10 cm, lit by overhead fluorescent tubes and shielded by dark curtains. Overhead digital video-camera records motion of individual zebrafish, with real-time object tracking at 5 Hz achieved using a desktop computer running MATLAB. Video frames and trajectory data are stored for subsequent analysis

The setup and apparatus for this study is described by Butail et al. (2014). Experimental subjects were monitored in a $120\times 120\times 20$ cm tank, supported on an aluminium frame, with a water depth of 10 cm (see Fig.1). The side length of the tank was thus approximately equal to 40 BL. The surface of the tank was covered with a white contact paper to enhance the contrast for automated tracking. Video recording was accomplished using a Microsoft LifeCam (USB interfaced) camera mounted 150 cm above the water surface, providing a single overhead video feed at a resolution of $640 \times 480$ pixels at 5 fps. At this resolution, the location of a fish was represented by approximately 20–50 pixels of each frame. Illumination was provided by diffused overhead lighting from four 25 W fluorescent tubes (All-Glass Aquarium, preheat aquarium lamp, UK). Video image analysis and real-time multi-target tracking was achieved using software developed in MATLAB (R2011a, Mathworks), sampled at 5 Hz on a 2.5 GHz dual-core Intel desktop computer with 3 Gb RAM (detailed description of tracking presented by Butail et al. (2013).

Experimental data consisted of individual video frames and trajectories consisting of two-dimensional position $\mathbf{x}_t = [x, y]_t$, with the origin at the centre of the tank, using a Cartesian coordinate system, and instantaneous velocity $\mathbf{v}_t = [\dot{x}, \dot{y}]_t$ computed using a Kalman filter such that the mean square error is continuously minimised. A full description of the Kalman filter and the associated measurement model used for velocity estimation can be found in the supplementary material of Butail et al. (2013). Fish were thus tracked as point masses, with heading information reconstructed from the velocity vectors. We expect analogous results to be obtained via the method for heading reconstruction presented in Gautrais et al. (2009). Future work will be performed using full shape tracking technique described in Bartolini et al. (2014) to assess the accuracy of different approaches for heading estimation from velocity data.

### Experimental procedure

A total of ten experimental observations were used for this investigation, each tracking the free-swimming trajectories of a different, experimentally naive, individual randomly selected from the population. Fish were removed from the holding tank using a small hand net and released into the experimental tank. Each observation was preceded with ten minutes of habituation time allowing the fish to swim freely and acclimatise to the novel environment of the experimental tank, as reported by Wong et al. (2010). The motion of each fish was recorded for 5 min (300 s), sampled at 5 Hz producing 1,500 position and velocity data samples per individual. Automated tracking was performed on video frame data in real-time, with minor adjustments made after each observation to repair missing trajectory points.

### Data extraction and pre-processing

Data for each observation was stored both as a sequence of image frames and a unique comma-separated data file containing the frame number and 2-dimensional position $\mathbf{x}_t$ and velocity data $\mathbf{v}_t$. The instantaneous speed $u_t$ at time $t$ was calculated from the vector norm of the velocity, with $u_t = \Vert \mathbf{v}_t\Vert$. The turning speed at a given time step $\omega _t$ is approximated using the (signed) angle turned between consecutive time steps $t \rightarrow t+\varDelta t$, to calculate a forward (angle) difference as follows:

1 $$\begin{aligned} \omega _t = \mathrm{sgn }\left[ \mathbf{v}_{t+\varDelta t} \times \mathbf{v}_t\right] _{z} \frac{1}{\varDelta t} \cos ^{-1} \left( \frac{\mathbf{v}_{t+\varDelta t} \cdot \mathbf{v}_t}{\Vert \mathbf{v}_{t+\varDelta t}\Vert \Vert \mathbf{v}_t \Vert }\right) \end{aligned}$$

where $\left[ \cdot \right] _z$ denotes the $z$-component of the cross product whose sign provides the turning direction (anti-clockwise positive). The time step $\varDelta t = 1 / f_s$ is the reciprocal of the sampling frequency ($f_s = 5$ Hz). Note that this definition of $\omega _t$ is consistent with that in Gautrais et al. (2009), and associates fish turning speed with the rate of change of the orientation of the velocity vector.

The model described in this paper is designed to study swimming zebrafish, as opposed to additional locomotory patterns, such as *freezing* or *thrashing* near obstacles (boundaries) as defined by Bass and Gerlai (2008). To obtain suitable data representing swimming behaviour, we used a simplified version of a method described by Kopman et al. (2012), pre-processing the ten raw observation data sets (denoted $F_1\ldots F_{10}$) to extract data segments (60 s) of equal duration in the following way:

1. Raw (speed) data was initially smoothed with a moving average window of 3 samples, then segmented such that contiguous segments are isolated when fish is moving with a speed above the threshold $u_\text {min} = 1$ BL s$^{-1}$. The original (unsmoothed) data is subsequently used for the steps that follow.
2. If the duration between consecutive segments was less than a time threshold $\tau _s = 2$ s, the two segments were joined so that fish were regarded as *not* swimming only if the duration of the speed dropping below threshold, $u_\text {min}$, exceeded $\tau _s$.
3. Resulting data segments were subdivided into intervals of equal length $\tau _l$ representing a continuous time-series of swimming data from an individual fish. Segments shorter than $\tau _l$ were discarded such that we obtain continues data segments of equal duration.

The isolated swimming data segments are denoted $S_1\ldots S_n$ and provide the data used for subsequent model development. Subsequent parameter estimation was carried on individual segments with average parameter sets calculated for each individual fish [$F_1\ldots F_{10}$] based on the swimming data isolated for each observation.

### Numerical implementation

Although an exact solution to the generalised O-U equation we will use in (7) exists, given by Gillespie (1996), nonlinearities in our model hamper its use. In order to calculate discretised solutions to the model SDEs in (8), expressed in the form $\hbox {d}X_t = a(X_t)\hbox {d}t+b(X_t)\hbox {d}W_t$, we therefore employed the Euler-Maruyama method, approximating the true solution $X$ with a Markov chain $M$ where

2 $$\begin{aligned} M_{t+\varDelta t} = M_t + \alpha (M_t)\varDelta t + \beta (M_t)\varDelta W_t \end{aligned}$$

Here, $\varDelta t$ is the time step duration and $\varDelta W_t$ are i.i.d. normal random variables with mean equal to zero and variance $\varDelta t$ (Kloeden and Platen 1992).

Simulated trajectories in this work were realised by iterating a random walker (RW) path in the plane, using numerically computed values of speed $U_t$ and turning speed $\varOmega _t$ over the interval $t=0\rightarrow T$ in time steps of duration $\varDelta t$. At each iteration $t$, the current heading direction $\phi _t$ was updated according to the value of $\varOmega _t$ and the walker moved forwards by a distance $U_t\varDelta t$. Specifically, heading directions were calculated as follows:

3 $$\begin{aligned} \phi _{t} = \left( (\phi _{t-\varDelta t} + \pi + \varOmega _t \varDelta t) \hbox { mod }2\pi \right) - \pi \end{aligned}$$

where shifts by $\pi$ and modulo operator ensure the heading angle varies smoothly in $[-\pi ,\pi ]$. The position $\mathbf{x}_t$ was subsequently updated at each time-step using

4 $$\begin{aligned} \mathbf{x}_{t} = \left[ x, y\right] _t = \left[ x, y\right] _{t-\varDelta t} + \left[ \cos \phi _t, \sin \phi _t \right] U_t \varDelta t \end{aligned}$$

and the corresponding velocity $\mathbf{v}_t$, computed by the backwards difference formula

5 $$\begin{aligned} \mathbf{v}_{t} = \left( \mathbf{x}_t - \mathbf{x}_{t-\varDelta t}\right) / \varDelta t \end{aligned}$$

For all simulations, the initial positions of random walkers $\mathbf{x}_0$ were randomly distributed within the simulation arena with uniformly distributed heading angles [$-\pi ,\pi$] and initial speed and turning speed $U_0=\mu _u$, $\varOmega _0=0$. The simulation time-step was set equal to the experimental sample rate such that $\varDelta t=0.2$ s.

Following the calculation of both $f_W$ and $f_c$ at a given time step $t$ (discussed in §4), the updated values of $U_t$ and $\varOmega _t$ were found using the method in (2), with values of $\varOmega _t$ restricted in the range $\pm 15\,\hbox {rad\,s}^{-1}$ in accordance to the observed maximum angular speed.

## Experiments

**Fig. 2:**
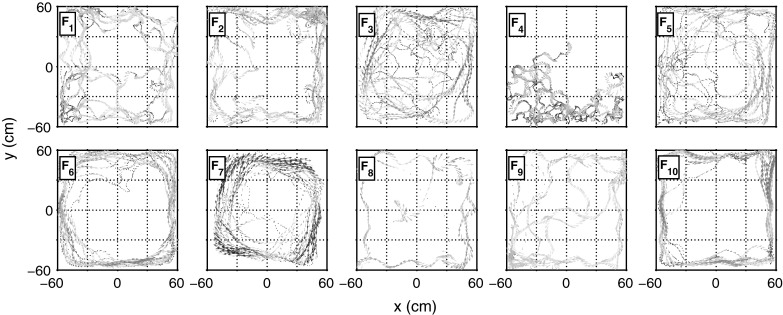
Raw trajectory data captured from 10 zebrafish (velocity plots). Observations were made for 5 min per individual fish, swimming in a shallow, square tank ($120\times 120\times 20$ cm, 10 cm water depth). Automated visual tracking recorded the planar position of the fish $\mathbf{x}_t$ within the tank at 5 Hz, calculating sample velocity $\mathbf{v}_t$ using a Kalman filter. The heading direction and magnitude of velocity are indicated by *coloured arrows*, increasing from *blue* to *red*. Swimming data was extracted from all fish apart from $F_4$ and $F_8$ which were found to swim erratically with long periods of freezing

The swimming speeds $u$ of 10 individuals were found to range from extended stationary periods, up to maximum speeds of approximately 31 cm s$^{-1}$, corresponding to just over $10$ body lengths (BL) per second (1 BL $\approx$ 3 cm). The average speed across the entire population was 3.53 BL.s$^{-1}$ with a standard deviation of 2.48 BL s$^{-1}$. Fish trajectories, shown in Fig. 2, were found to vary between individuals: some performed erratic, tightly winding manoeuvres (e.g. $F_4$); whilst others had more fluid trajectories which explored an increased area of the tank (e.g. $F_1, F_3, F_5$). We also found strong, persistent wall-following behaviour *(thigmotaxis)* (e.g. $F_6, F_7, F_{10}$) along with extended periods of freezing or thrashing behaviour (e.g. $F_2, F_8, F_9$).

### Swimming trajectory analysis

Using the data segmentation process described earlier, we isolated 28 segments of swimming data, each 1 min in duration, representing 8 out of the 10 raw observations, where $F_4$ and $F_8$ failed to produce data which met all filtering criteria for swimming. The pre-processing method was also found to eliminate periods of excessive thrashing, characterised by large amplitude fluctuations in $\omega _t$. Speed and turning speed time series data $u_t$ for each segment, labelled consecutively from $S_1$ to $S_{28}$, are shown in-situ with the corresponding raw data in supplementary Figs. S1 and S2.

**Fig. 3:**
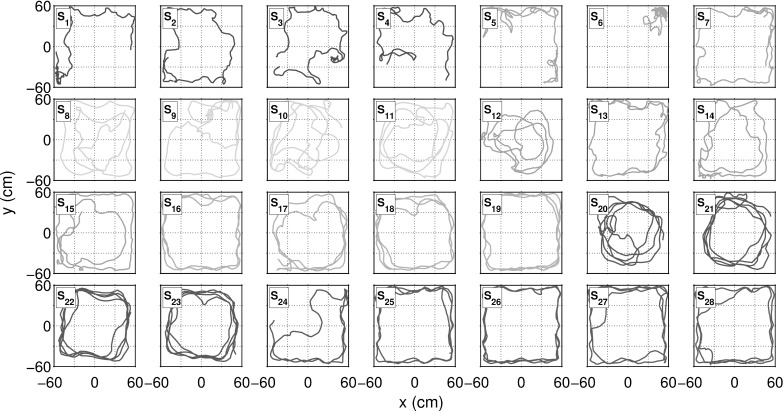
Isolated swimming segment trajectories. Denoted $S_1\ldots S_{28}$, each trajectory, of 60 s in duration, are coloured according to the originating fish observation ($F_1\ldots F_{10}$), where qualifying data has been extracted from 8 out of 10 unique individuals ($F_4,F_8$ failed to provide sufficient data)

Analysis of the 28 min of data, isolated for active swimming behaviour for 8 fish, yielded a (increased) combined mean swimming speed of $4.65\,\hbox {BL\,s}^{-1}$ with (reduced) standard deviation of $2.01\,\hbox {BL\,s}^{-1}$; in good agreement with other studies e.g. Fuiman and Webb (1988), Plaut (2000) which report the mean speed of zebrafish groups to be $\sim 13\,\hbox {cm.s}^{-1} (4.3\,\hbox {BL\,s}^{-1}$). Mean turning speed was found to be $-0.02\,\hbox {rad\,s}^{-1}$ with a standard deviation of $1.30\,\hbox {rad\,s}^{-1}$, indicating negligible turning direction preference, with the convention that positive turning speeds represent turns to the *left*, negative to the *right*. Maximum and minimum values of turning speed were found to be $-14.73$ and $14.28\,\hbox {rad\,s}^{-1}$ respectively, suggesting a (global) absolute maximum turning speed $\hbox {max}(|\omega |) \approx 15\,\hbox {rad\,s}^{-1}$. Maximum turning speeds were found to be close to the upper limit detectable between consecutive samples at frequency $f_s$, where $\omega _{\mathrm {max}} = \pi f_s = 5\pi \approx 15.71\,\hbox {rad\,s}^{-1}$. Such high speed turns however are observed with very low frequency across filtered swimming segment data, with turns faster than $5\,\hbox {rad\,s}^{-1}$ accounting for less than 1% of all samples. Isolated swimming trajectory portraits (Fig. 3) display the variety of different characteristic behaviours described earlier. In particular, we observed strong wall-following behaviour which leads to an individual bias of the turning speed in the direction of rotation around the walls.

**Fig. 4:**
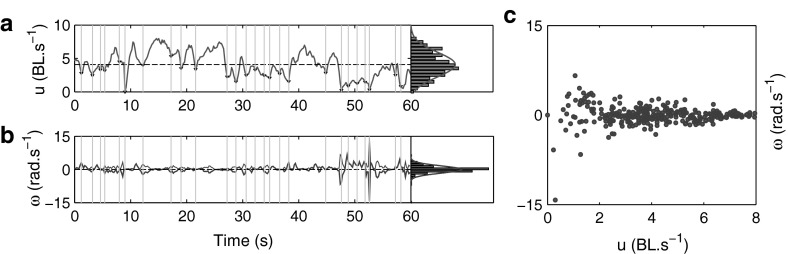
Example data from swimming segment $S_{9}$. **a** and **b** Time series data for speed $u_t$ and turning speed $\omega _t$ respectively, with associated relative frequency histograms and fitted normal distribution p.d.f (smooth *red*, *blue curves*). Vertical delineations in time-series plots indicate minima in $u_t$ with a prescribed minimum separation of 0.5 s (2 Hz). Identical *lines* in $\omega _t$ time-series highlight a temporal correlation where speed minima are generally associated with peaks in $|\omega _t|$ (thin *blue* trace). **c** Values of $\omega _t$ against $u_t$ indicating a correlation with increased probability of large turning speed $\omega _t$ values as $u_t$ decreases

During active phases of swimming, the instantaneous speed $u_t$ of an individual was found to have a well-defined mean, yet highly variable with rapid bursts of forward thrust corresponding to the natural tail-beat frequency between $0.5$ and $2$ Hz, identified through spectrographic analysis of the time series data. Additionally, we observed strong correlations between the time onset of bursts in turning speed with those in instantaneous speed (Fig. 4a, b). Strong correlations were also found between the magnitude of $u_t$ and the variance of $\omega _t$ (Fig. 4c). This characteristic feature may be associated with momentum conservation from tail-beating, which simultaneously provides both axial force and torque, to produce forward and turning motion, respectively (Sfakiotakis et al. 1999).

The distribution of instantaneous speed $u_t$ was found to be approximately normal with a natural truncation at $u = 0$ cm s$^{-1}$. Individually parameterised Gaussian density functions therefore yield a good approximation to the distributions of $u_t$ (Fig. 4a). Analysis of $\omega _t$ (Fig. 4b) similarly indicated that a normal probability density function provides reasonable approximations to experimental data, with a mean close to zero. In general however, the distributions of $\omega _t$ were found to be more sharply peaked than a Gaussian, with heavy tails due to a low proportion of extreme values of turning, both left ($\omega \gg 0$) and right ($\omega \ll 0$), resulting in larger estimates of the standard deviation and flattening of the corresponding Gaussian probability distribution function (pdf). As such, a normal distribution is found to be appropriate when the sample standard deviation $\hat{\sigma }_\omega < 1.5\,\hbox {rad\,s}^{-1}$. Above this value, a normal distribution fails to capture both the sharp peak around the mean, and the finite probability of rapid changes in heading. In the model we describe later in this section, the speed process $u_t$ is assumed to be a stationary Gaussian process, whilst the turning speed $\omega _t$ is assumed to be a Gaussian process with varying variance. Additionally, these processes are coupled such that we recover both the observed cross-correlation and a correction to the distribution of the turning speed.

Autocorrelation functions (ACF) for both $u_t$ and $\omega _t$ were computed for all segments, revealing consistent and well-defined properties. Both processes display a large positive correlation at short time-lags which decays with an approximately exponential envelope with varying degrees of noisy oscillation. We quantified the decay rate of the ACF by considering the lag-one autocorrelation coefficient $r_1$ of discretely sampled signal $\{X_k\}_{k=1}^N$, given by

6 $$\begin{aligned} r_1 = \frac{\sum ^{N-1}_{k=1} (X_k - \bar{X})(X_{k+1}-\bar{X})}{\sum ^{N}_{k=1} (X_k-\bar{X})^2} \end{aligned}$$

where $\bar{X}$ is the sample mean.

**Fig. 5:**
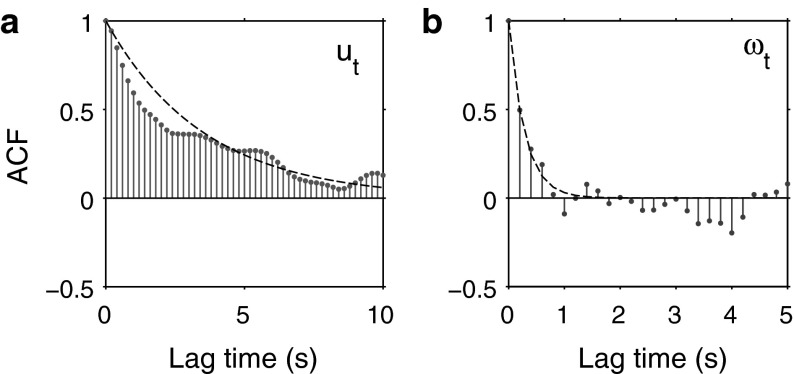
Autocorrelation of $S_9$ speed and turning speed. **a** $\hbox {ACF}_u$ up to 5 s of lag with exponential approximation (*black dashes*) $\hbox {ACF}_u \approx \exp {(-t/\tau _u)}$, $\tau _u = 3.54$ s (b) $\hbox {ACF}_\omega$ up to 5 s of lag with $\hbox {ACF}_\omega \approx \exp {(-t/\tau _\omega )}$, $\tau _\omega = 0.29$ s. Autocorrelation half-life for either process is thus approximated by $\tau \hbox {ln}(2)$, where $\tau$ is the one-step autocorrelation coefficient

We considered the associated correlation time $\tau$ for the ACFs, where $\tau = -\varDelta t/\hbox {ln}(r_1)$ was used to parameterise an exponential function $\hbox {ACF}_{est} = \exp (-t/\tau )$ estimating the autocorrelation decay envelope. Across the majority of segments, a exponential approximation provides a good estimate for both $\hbox {ACF}_u$ and $\hbox {ACF}_\omega$ (example shown for segment $S_9$ in Fig. 5). The average autocorrelation half-life ($\tau \,\hbox {ln}\,2$) for $u_t$ and $\omega _t$ across segments, were found to be approximately 1.37 and 0.28 s, respectively.

## Modelling

Our analysis of zebrafish trajectory data suggests that a model in which speed is held constant may be insufficient to describe the individual and collective motion of zebrafish and other similar species of small, schooling fish. Compared with the smooth, continuous motion of larger fish, for example *Kulia mugil* (BL $\approx 20$ cm) modelled by Gautrais et al. (2009), the variance in swimming speed for smaller zebrafish (BL $\approx 3$ cm) is large. Many factors influence the range and fluctuations of swimming speed, with drag being the primary physical component, scaling with the square of the wetted surface area. In the presence of viscous drag, with a flow regime dependent on the specific Reynolds number, different aquatic species have evolved a range of swimming styles as described by Sfakiotakis et al. (1999). Specifically for zebrafish, their small size and tail morphology results in a burst-and-coast mode of locomotion, which has been found to be more efficient than a continuous swimming style, as discussed by Weihs (1972), Muller et al. (2000).

The key features of both signals considered independently, instantaneous speed $u_t$ and turning speed $\omega _t$, are well approximated by stationary, Gaussian processes with exponentially decaying autocorrelation functions. These observations lead us to adopt the formalism of a continuous-time autoregressive system with two independently parameterised Gaussian processes for $u_t$ and $\omega _t$. The basis of our model is thus an extension of the PTW model presented by Gautrais et al. (2009) in which the turning speed $\varOmega _t$ of a simulated random walker is modelled by a signal $S_t$, a stochastic process of the Ornstein–Uhlenbeck (O-U) family with the general form

7 $$\begin{aligned} \hbox {d}S_t = \theta (\mu -S_t)\hbox {d}t+\sigma \hbox {d}W_t \end{aligned}$$

with equilibrium (relaxation) value $\mu$, rate of mean-reversion $\theta$, and variance $\sigma$ of the standard Wiener process $W_t$. As $t\rightarrow \infty$ the stationary solution of this SDE has a Gaussian distribution with a mean and variance approaching $\mu$ and $\sigma ^2/2\theta$, respectively (Gardiner 2009).

In our extended model, we consider two coupled stochastic equations, describing both the speed $U_t$ and turning speed $\varOmega _t$ as follows:

8a $$\begin{aligned}&\hbox {d}U_t = \theta _u (\mu _u-U_t)\hbox {d}t + \sigma _u \hbox {d}W_t \end{aligned}$$

8b $$\begin{aligned}&\hbox {d}\varOmega _t = \theta _\omega (\mu _\omega + f_W-\varOmega _t)\hbox {d}t + f_c \hbox {d}Z_t \end{aligned}$$

where the SDEs for speed (8a) and turning speed (8b) have the same general form presented earlier in (7), describing random processes exhibiting noisy relaxation to a mean $\mu$ and, importantly, with an exponentially decaying ACF with rate $\theta = 1/\tau$. Additive noise is driven by the Wiener processes d$W$ and d$Z$, with variances parameterised by $\sigma _u^2$ and $f_c^2$, acting on the *differentials* of position and heading respectively.

In order to recover the observed correlation between the magnitude of $u_t$ and variance of $\omega _t$ (e.g. Fig 4c), we introduce the function $f_c = f_c(U_t,\sigma _\omega ,\sigma _0,\mu _u)$, which couples the two processes such that the variance of the random fluctuations of turning speed $\varOmega _t$ depends on the speed $U_t$. Wall (boundary) avoidance is achieved by incorporating a second function $f_W = f_W(\phi _W,d_W)$ in (8b), which models the tendency of fish to avoid collisions with the tank walls, where $d_W$ and $\phi _W$ are the distance and angle of projected collision with a boundary, given the velocity at a given time step. The features encapsulated by these two additional functions $f_W$ and $f_c$, including the estimation of all model parameters, are described in what follows.

### Selection of wall avoidance function $f_W$

Following the approach of Gautrais et al. 2009, the term $f_W$ is constructed to reflect the observed turning speed distribution as a function of *distance* $d_W$ (Gautrais et al. 2009), or *time* $t_W$ (Gautrais et al. 2012) with which a projected collision with the boundary would occur given the current position and velocity of the fish. To quantify this effect, we calculate the distribution of a ‘wall-corrected’ value of the turning speed $\omega _c$ which is positive when the direction of a turn is away from the collision boundary and vice versa, such that

9 $$\begin{aligned} \omega _{c_t} = {\left\{ \begin{array}{ll} \vert \varOmega _t \vert &{} \text {if } \hbox {sgn}(\varOmega _t) = \hbox {sgn}(\phi _{W_t})\\ -\vert \varOmega _t \vert &{} \text {otherwise} \end{array}\right. } \end{aligned}$$

The direction of the induced turn characterised by $f_W$ is therefore prescribed by calculating the (signed) angle $\phi _W$ between the current heading and the normal at the point of collision. The plots in Figs. S3 and S4 display show $\omega _c$ calculated from each segment, as a function of $d_W$ and $t_W$ respectively.

Based on the fact that the turning dependence was very similar for both $d_W$ and $t_W$, we choose the form

10 $$\begin{aligned} f_W(\phi _W,d_W)&= \hbox {sgn}(\phi _W) A \exp (B d_W) \end{aligned}$$

Retaining the formalism of the general O-U process (7), $f_W$ provides a smooth, exponentially increasing bias to the equilibrium value of the $\varOmega$ turning speed process, in the direction which *increases* the projected distance (or time) to collision. Parameters $A$ and $B$ control the strength and decay of the repulsion $f_W$ and are estimated from experimental data as described in the following section (Parameter estimation). A similar analysis on the effect of wall boundaries on speed regulation suggested that speed is only marginally influenced by the wall distance (Fig. S5, S6). For this reason we opted not to include a functional dependence on either $d_W$ or $t_W$ for the speed process in (8a) as we have for turning speed.

As the repulsive turning effect supplied by $f_W$ does not implicitly prevent trajectories from crossing the simulated boundaries, we also include an additional *hard*-boundary condition. Our simple strategy is to model wall encountering events as fully inelastic collisions, such that the speed $U_t$ of a random walker passing through a boundary at time $t$ is set to zero, leaving its position unchanged from the previous time step, so that $\mathbf{x}_t \leftarrow \mathbf{x}_{t-\varDelta t}$.

In order to replicate experimental conditions, simulated trajectories were modelled in a bounded, rectangular arena. However, a finite, rectangular simulation arena presents discontinuous boundaries at each corner which must be smoothed to prevent competing repulsion by perpendicular walls near the vertices from creating singularities for point-like random walkers. By rounding the edges of the simulation arena with quarter-circles of radius $R_c$, we avoid unrealistic turning behaviour at the corners of the tank, where a value of $R_c=10$ cm was found to sufficiently reduce undesirable artefacts in these regions.

### Selection of coupling function $f_c$

**Fig. 6:**
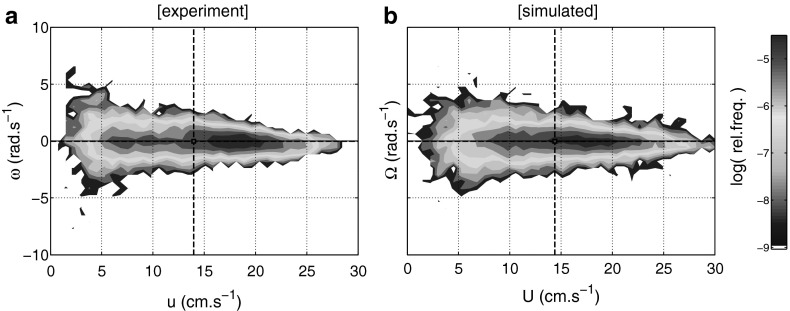
Speed and turning speed cross correlation—achieved by coupling SDEs. **a** Log-frequency density contours of experimental data from $S_1\ldots S_{28}$ indicate the narrowing of distribution of $\omega$ as $u$ increases. **b** Log-frequency density contours of simulated data. By coupling the SDEs for $\varOmega _t$ and $U_t$ using function $f_c$ we recover a comparable joint distribution (28 trajectories each of equal 60 s, with O-U parameters calibrated on corresponding experimental segments, using a constant value of $\sigma _0 = 12\,\hbox {rad~s}^{-1}$. *Black dashed lines* indicate mean values of speed $\bar{u}_t$ and turning speed $\bar{\omega }_t$ for experimental and simulated data $\bar{U}_t$ and $\bar{\varOmega }_t$ on respective plots

The joint distribution of experimental data on $u_t$ and $\omega _t$ was found to be highly asymmetric, with a much larger proportion of faster turns occurring at low speeds. A composite log-density plot of cross-correlated data is shown in Fig. 6a.

Fast turning speeds are associated with lower forward speeds due to mechanics of fish locomotion. To model this relationship we therefore require a coupling between the two SDEs in (8a) and  (8b), such that we obtain similar distributions of speed $U_t$ and turning speed $\varOmega _t$ to those observed in the experiments. Specifically, we substitute the variance parameter $\sigma$ in the general O-U process description in (7) with a function $f_c$ in the form of an exponential decay with respect to the speed $U_t$. Due to heavy-tails of the observed turning speed distribution, calibration using a maximum likelihood estimation (MLE) following the method described by van den Berg (2011) (assuming a standard O-U process with a normal distribution) will tend to overestimate $\sigma$ from source data $\omega _t$ and overly increase the variability of the simulated turning speed process $\varOmega _t$. We account for this by choosing $f_c$ such that

1. When $U_t$ approaches zero, the function returns the upper bound, say $\sigma _0$ on the variance of the turning speed (to be estimated from the experimental observations), or equivalently
  11 $$\begin{aligned} \lim _{U_t \rightarrow 0}f_c = \sigma _0,\quad \sigma _0 > \sigma _\omega \end{aligned}$$
2. The function approaches zero as $U_t$ goes to infinity.
3. The function returns a value dependent on the variance $\sigma _\omega$ of the turning speed when $U_t$ is equal to the average speed $\mu _u$, which will be estimated so as to better capture the observed experimental distribution (see Fig. 6).

A simple exponential function $f_c$, parameterised by the mean speed $\mu _u$, variance $\sigma _\omega$, and a fixed maximum value $\sigma _0$ (see 4.3) satisfies all of these criteria, choosing

12 $$\begin{aligned} f_c(U_t,\sigma _\omega ,\sigma _0,\mu _u) = \sigma _0\left( \frac{2 \sigma _0}{\sigma _\omega }\right) ^{-\frac{U_t}{\mu _u}} \end{aligned}$$

As demonstrated in Fig 6b, this function allows to recover a distribution of $U_t$ and $\varOmega _t$ which are highly comparable with experimental data.

### Parameter estimation

**Fig. 7:**
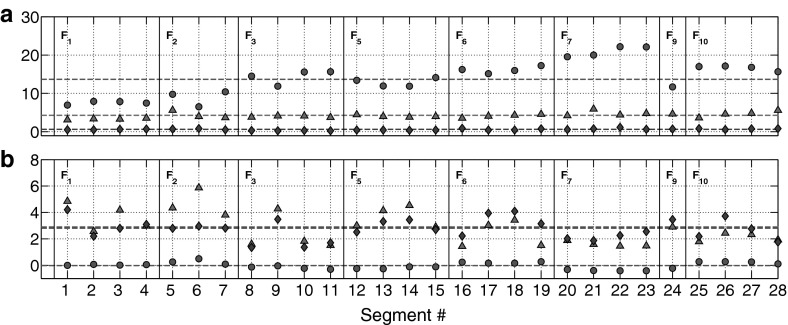
Comparison of MLE calibrated parameters for individual data segments, grouped by parent fish ID. $\mu$ (*red circles*), $\theta$ (*blue diamonds*) and $\sigma$ (*magenta triangles*). *Dashed lines* indicate the respective weighted mean parameter values. **a** Parameters $[\mu _u,\theta _u,\sigma _u]$ for speed data $U_t$. **b** Parameters $[\mu _\omega ,\theta _\omega ,\sigma _\omega ]$ for turning speed data $\varOmega _t$

The six parameters $[\mu ,\theta ,\sigma ]_{u,\omega }$ for individual segments $S_1 \ldots S_{28}$ were found using MLE, following the method of van den Berg (2011), under the assumption that source data $u_t$ and $\omega _t$ both come from independent Gaussian processes described by (7). Equivalent parameters corresponding to the individual fish $F_1$, $F_2$, $F_3$, $F_5$, $F_6$, $F_7$, $F_9$, $F_{10}$ were subsequently calculated by averaging over the corresponding segments values extracted from each fish. The relationship between segments to parent observation data and MLE calibrated parameter values are shown in Fig. 7, indicating generally consistent parameters across individuals. We also calculated a global set of mean parameters $[\tilde{\mu }_u,\tilde{\theta }_u,\tilde{\sigma }_u]$ (speed) and $[\tilde{\mu }_\omega ,\tilde{\theta }_\omega ,\tilde{\sigma }_\omega ]$ (turning speed), computed by weighting the individual means by the number of segments isolated for each fish, e.g.

13 $$\begin{aligned} \tilde{\mu }_u = \frac{\sum _{i=1}^{10} n_i \mu _u[F_i]}{\sum _{i=1}^{10} n_i} \end{aligned}$$

where $n_i$ is the number of segments isolated for each fish $F_i$. The values calculated in this way are found below, and also in Table 1 alongside parameter values for each individual fish. They are:

14 $$\begin{aligned} \tilde{\mu }_u&= 14.02 \hbox { cm\,s}^{-1} (4.67 \hbox { BL\,s}^{-1})&\nonumber \\ \tilde{\theta }_u&= 4.21 \hbox { s}^{-1}&\nonumber \\ \tilde{\sigma }_u&= 0.59 \hbox { cm\,s}^{-1}&\nonumber \\ \tilde{\mu }_\omega&= -0.02 \hbox { rad\,s}^{-1}&\nonumber \\ \tilde{\theta }_\omega&= 2.74 \hbox { s}^{-1}&\nonumber \\ \tilde{\sigma }_\omega&= 2.85 \hbox { rad\,s}^{-1}&\end{aligned}$$

**Table 1:** Mean parameter values for each fish $F_1\ldots F_{10}$, calculated from 28 isolated data segments.

		${F}_{1}$	${F}_{2}$	${F}_{3}$	${F}_{4}$	${F}_{5}$	${F}_{6}$	${F}_{7}$	${F}_{8}$	${F}_{9}$	${F}_{10}$	
	# of segments	4	3	4	0	4	4	4	0	1	4	*(28)*
$u(t)$	$\mu _u$	7.555	8.899	14.406	N/A	12.841	16.135	20.964	N/A	11.719	16.642	***14***.***02***
	$\theta _u$	0.592	0.661	0.298	N/A	0.426	0.633	0.771	N/A	0.717	0.741	***0***.***59***
	$\sigma _u$	3.350	4.447	3.981	N/A	4.086	4.118	4.810	N/A	4.651	4.689	***4***.***21***
$\omega (t)$	$\mu _\omega$	0.028	0.280	-0.183	N/A	-0.187	0.206	-0.384	N/A	$-0.221$	0.221	$-$ ***0***.***02***
	$\theta _\omega$	3.077	2.858	1.971	N/A	2.996	3.347	2.175	N/A	3.456	2.606	***2***.***74***
	$\sigma _\omega$	3.651	4.676	2.304	N/A	3.649	2.355	1.599	N/A	2.914	2.128	***2***.***85***

Weighted averages providing a common (global) set of parameters are indicated in bold

**Fig. 8:**
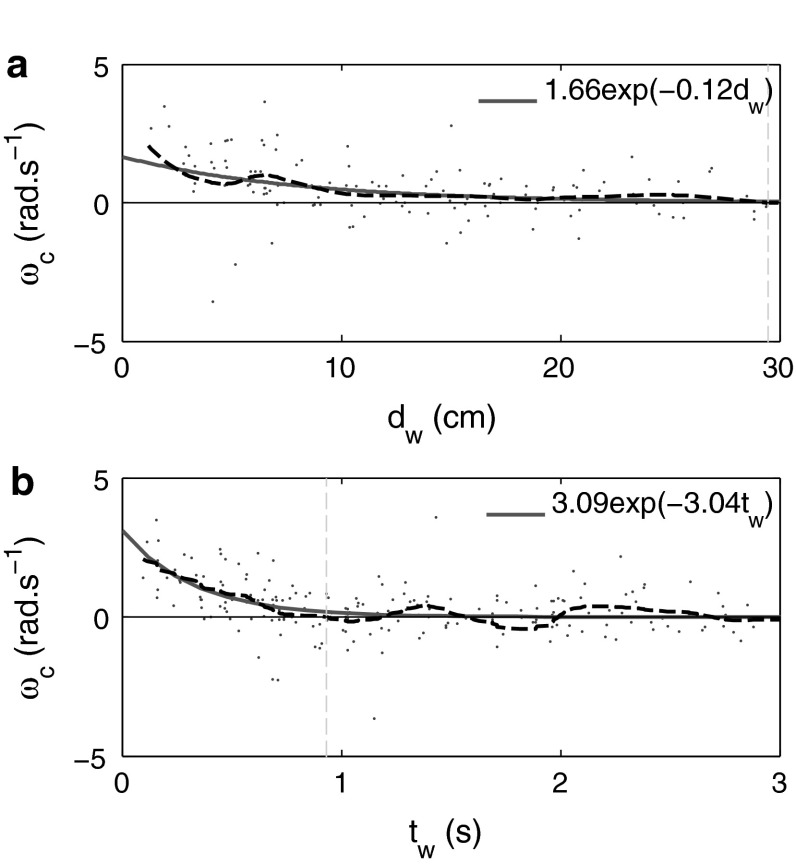
Effect of boundary on turning speed with respect to the projected distance and time to collision with tank wall on segment $S_{27}$ data. Non-parametric (LOESS) regression (*black dashed lines*) highlight increased turning to *avoid* collisions ($\omega _c > 0$). **a** Wall corrected turning speed $\omega _c$ is plotted as a function of the projected distance $d_W$ to impact with a wall. Exponential function $\omega _c = A_de^{B_d d_W}$ (*red curve*) approximates a ‘soft’ repulsion as a function of projected distance with fitted parameters $A_d=2.14\,\hbox {rad\,s}^{-1}$,$B_d=-0.16$ cm. **b** wall-corrected turning speed $\omega _c$ is plotted as a function of the projected time $t_W$ to impact with a wall. Exponential function $\omega _c = A_t e^{B_t t_W}$ (*red curve*) approximates repulsion as a function of projected distance with fitted parameters $A_t =3.00\,\hbox {rad\,s}^{-1}$, $B_t=-2.98$ s

The parameters $A$ and $B$ of the wall repulsion function $f_W$ in (10), specifying the strength and range respectively, were found by calculating the distance $d_W$ which a fish, at a given sample position, is projected to collide with the tank wall, given its current velocity. For each data sample, we constructed the wall-corrected turning speed $\omega _c$ as described in (9) by modifying the sign of $\omega _t$ to reflect turning either towards or away from the boundary. By plotting sample values of $\omega _c$ against the projected distance $d_W$, or time $t_W$ to wall collision, we observe a distinct bias for turns which favour impact avoidance ($\omega _c > 0$) as $d_W$ decreases. To approximate this bias, we used a non-parametric locally weighted least squares (LOESS) model (Gijbels and Prosdocimi 2010), fitting the resulting interpolation with a parametric exponential function. Using this method, averaging across swimming segments, we estimated the parameters $A$ and $B$ in (10) determining the repulsive (*turning*) effect of the boundary on turning speed as a function of $d_W$. An example of the two-step interpolation for segment $S_{27}$ is shown in Fig. 8a. By considering only segments with which a reasonable fit could be obtained $[S_2, S_3, S_4, S_{6-11}, S_{13}, S_{16}, S_{17}, S_{19}, S_{20}, S_{22}, S_{23}, S_{25-28}]$, we found average parameter values $A=2.25\pm 0.70\,\hbox {rad\,s}^{-1}$ and $B = -0.11\pm 0.04$ cm. For completeness, we also calculated parameters values for $A$ and $B$ for a time-to-collision dependence $t_W$ (see Fig. 8b), averaging over segments $[S_4,S_7,S_{8-11},S_{13},S_{16},S_{18-28}]$ to give $A = 2.25 \pm 0.62\,\hbox {rad\,s}^{-1}$ and $B = -1.68\pm 0.53$ s. From our analysis we found no compelling evidence supporting a stronger functional dependence of turning speed on either $d_W$ or $t_W$ thus we proceeded with a wall-avoidance function dependent solely on projected collision distance $d_W$ and the collision angle $\phi _W$. Graphical depictions of the dependence of $\omega _c$ and $u_t$ on projected collision distance $d_W$ and time $t_W$ for all segment data can be found in supplementary Figs. S3–S6.

Heuristically, we found that a magnified value of $A$ was required to produce turning behaviour comparable to experimental observations. After many realisations of random walkers with various calibrations, we chose to increase the above value of $A$ by a factor of 3 such that we simulate all random walkers with $A = 6.75\,\hbox {rad\,s}^{-1}$ and $B = -0.11$ cm, calculating $f_W(d_W,\phi _W)$ from Eq. (10). This discrepancy results either from interpolating $f_W$ with an insufficient number of data points close to the boundaries, or the compensation required to account for oversimplification of the wall avoidance model.

Finally, we estimated the saturation parameter $\sigma _0$ in (12) so as to obtain a similar correlation between simulated values of $U_t$ and $\varOmega _t$, with consideration given to the range and distribution of observed turning speeds. Although the absolute maximum value was found to be $\approx 15\,\hbox {rad\,s}^{-1}$, the distribution of experimental $\omega _t$ is heavy-tailed such that values of $\vert \omega \vert > 10\,\hbox {rad\,s}^{-1}$ account for less than 0.2% of recorded samples. We therefore prescribed a reasonable upper bound to the function $f_c$ by finding $\sigma _0$ such that the probability of generating turning speed values $\varOmega _t$ from (8b) below a speed of approximately $10\,\hbox {rad\,s}^{-1}$, is within the 2-sigma ($\sim 95$%) confidence interval i.e. $2\hat{\sigma } \approx 10\,\hbox {rad\,s}^{-1}$, where $\hat{\sigma }$ is the long term variance of the output process. The variance $\hat{\sigma }^2$ of $\varOmega _t$ was estimated by assuming a general O-U process where, for the saturation variance $\sigma _0$ we have $\hat{\sigma }^2 = \sigma _0^2/2\tilde{\theta }_\omega$ (Gardiner 2009). Using the maximum $\omega _t$ as an estimate of two standard deviations ($2\hat{\sigma }$), we obtain the formula

15 $$\begin{aligned} \sigma _0 = \frac{{\hbox {max}(\omega _t)\sqrt{2\tilde{\theta }_\omega }}}{2} \end{aligned}$$

Using the values max($\omega _t)=10\,\hbox {rad\,s}^{-1}$ and $\tilde{\theta }_\omega =2.81\,\hbox {s}^{-1}$, the global average value from (14), we obtain the maximum variance parameter value $\sigma _0 \approx 12\,\hbox {rad\,s}^{-1}$. Accordingly, the resulting composite distribution of simulated values of $U_t$ and $\varOmega _t$ for all segment calibrations, shown in Fig. 6b, indicates a good approximation to the experimental distribution in Fig. 6a.

### Model consistency

Initial validation of the model was conducted by comparing the trajectories, and underlying metrics, of individual swimming segments to those of simulated random walkers with SDE parameters calibrated from the corresponding experimental segments. The remaining parameters were fixed globally across all segments using the values described earlier in this section, summarised in Table 2. A square simulation arena with side length $L=120$ cm was also defined to match the dimensions of the experimental tank.

**Table 2:** Global simulation parameters

Parameter description	Symbol	Unit	Value
Simulation time step	$\varDelta t$	s	0.2
Simulated tank (square) side length	$L$	cm	120
Rounded edge circle radius	$r_c$	cm	10
Maximum turning speed variance	$\sigma _0$	$\hbox {rad\,s}^{-1}$	12
Max. turning speed (cut-off)	n/a	$\hbox {rad\,s}^{-1}$	15
Wall avoidance function amplitude	$A$	$\hbox {rad\,s}^{-1}$	6.75
Wall avoidance function decay	$B$	cm	–0.11

Data simulated across a range of sample generation frequencies, 1000–5 Hz ($\varDelta t$ = 0.001–0.2 s) using identical stochastic processes $dW_t$ and $dZ_t$, indicated that trajectories and their underlying statistics (distributions, ACFs etc.) were sufficiently robust to increasing values of $\varDelta t$ over three orders of magnitude (see Fig. S7). Using a value $\varDelta t = 0.2$ s was therefore found to provide a good compromise between numerical accuracy and computational efficiency^1^, with a corresponding sample generation rate of 5 Hz matching that of the experimental acquisition frequency.

**Fig. 9:**
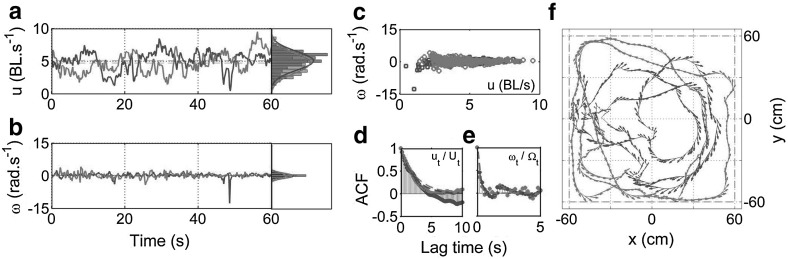
Comparison between simulated random walker for $S_{17}$ and experimental segment data. Data highlights the key metrics to compare swimming segment $S_{17}$ *(blue)* against those of a random walker (*red*), with parameters calibrated directly on the experimental segment. **a** Time series and distribution for speed data $U_t$. **b** Time series and distribution for turning speed data $\varOmega _t$. **c** Cross-correlation between $U_t$ and $\varOmega _t$. **d** Autocorrelation function $\hbox {ACF}_u$. **e** Autocorrelation function $\hbox {ACF}_\omega$. **f** Trajectory vector comparison

An example simulation, calibrated on segment $S_{17}$, is shown in Fig. 9, compared with the experimental data for both speed and turning speed. Simulated speed data (shown in red) yields a normal distribution which corresponded well with the experimental data (Fig. 9a). The speed autocorrelation function $\hbox {ACF}_u$ (Fig. 9d) was also in good agreement with that of the experimental source data, in particular capturing the initial decay prior to the zero crossing. Simulated turning speed data (blue), generated by a modified (coupled) O-U type process, was found to have a distribution which is more sharply peaked than a Gaussian process, and in good agreement with source data $\varOmega _t$ (Fig. 9b). Here, we note an additional effect of the SDE coupling, where $f_c$ restricts the probability of high speed turning to periods of reduced forward speed. A direct result of this is to produce a turning speed distribution with a sharper peak around the mean ($\mu _\omega \approx 0$), and with heavier tails such that extreme values of $\varOmega$ are found with low probability, but more often than would be produced by a normally distributed (unmodified) O-U process. The joint distribution of speed and turning speed (Fig. 9c) also presents a successful recovery of the experimental distribution, characterised by a narrowing of the turning speed distribution as speed increases. By appropriately coupling the SDEs via $f_c$ we therefore achieve both recovery of the cross-correlation between $u_t$ and $\omega _t$, and a favourable modification to the distribution of $\omega _t$ to match those observed experimentally. Similarly, for the corresponding autocorrelation $\hbox {ACF}_\omega$ (Fig. 9e), we are able to capture the characteristic exponential decay, with a rate very similar to the experimentally observed value. A final comparison is made in Fig. 9f, showing the resulting 60 s RW trajectory portrait and velocity vector plot, overlaid on the experimental source data.

To support the inclusion of the coupling function $f_c$ in our proposed model, we simulated comparable trajectories in the absence of coupling (fixed $\sigma _\omega$). Simulated realisations for representative experimental segments $S_3$ and $S_{17}$ are shown in Fig. S14, both with and without the coupling between $U_t$ and turning speed variance $\sigma _\omega$. In the uncoupled trials, we clearly fail to capture a reasonable estimate for the joint distribution of speed and turning speed (see column B in Fig. S14). Without $f_c$ to restrict the turning speed variance at high speeds, we find a more normal spread of $\varOmega _t$ which fails to capture the sharp peaks of the experimental distributions (column D). The one-way coupling between two processes should have no effect on the distribution and autocorrelation of speed data (columns B and E), however we also note that we do not find significant effects on the turning speed ACF (column F). Importantly, we find that trajectories produced by the coupled model appear to be qualitatively more consistent with experimental segment trajectories (column A). We note a higher propensity to enter longer lasting/long path length spiralling when the process are uncoupled, features which are reduced by the coupling as large turning speed variance (increased range in either direction) is reduced at high speeds—and therefore only available to the random walker at lower speeds where less distance will be covered during such a turn. We also note that decoupling the processes reduces the propensity to elicit wall-following behaviour when calibrated on segments exhibiting these phenomena (again due to the increase range of turning speeds when decoupled or conversely because, when coupled, the turning speed distribution is more sharply peaked around zero).

Plots for all segments (coupled model), comparing a single random walker realisation to experimental source data, can be found in the supplementary information (Figs. S8–S13). We also refer again to plots depicting the dependence of speed and wall-corrected turning speed on projected distance and time to boundary collision in supplementary Figs. S3–S6.

Further tests of model consistency are provided by comparing eight random walker trajectories, simulated using the averaged parameter values for individual fish $F_1$, $F_2$, $F_3$, $F_5$, $F_6$, $F_7$, $F_9$ and $F_{10}$ found in Table 1, to composite zebrafish trajectories from the corresponding experimental segments $S_1\ldots S_{28}$. Single random walker realisations, calibrated for each fish are shown in Fig. 10, simulated for a time $T=60n_s$ where $n_s$ is the number of segments isolated for each fish. We observe that broadly similar qualitative turning characteristics of each zebrafish are recovered, including the propensity for wall-following behaviour. From these simulations, we find that the model is able to effectively extract and reproduce trajectory data which closely approximates the swimming motion, and subtleties of individual fish, and also how the underlying statistics may be used to predict a form of ‘passive’ thigmotactic-like behaviour^2^. Specifically, the approximate ratio $\sigma _\omega / \theta _\omega$ is found to provide a good predictor of the observed thigmotactic-like behaviour that is well captured by the model. In order of increasing ratio, fish $F_6$, $F_7$ and $F_{10}$ exhibit the most consistent wall-following behaviour, with values of $\sigma _\omega / \theta _\omega < 1$. Consequently, fish which are found to spend a larger fraction of time away from the walls, for example $F_2$, $F_5$, $F_1$, in order of *decreasing* ratio, are found to have $\sigma _\omega / \theta _\omega > 1$.

**Fig. 11:**
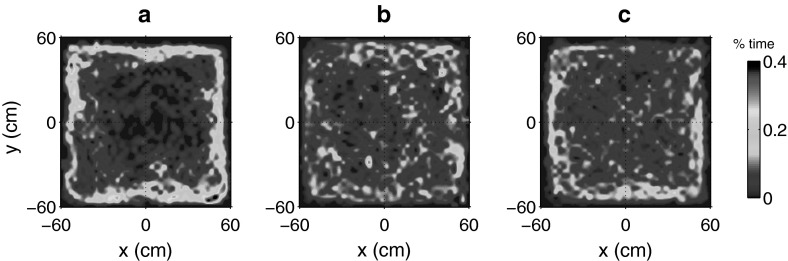
Trajectory density comparison between experimental and simulated data. Trajectory data in the form of density plots, with pixel *color* representing the percentage time spent at each location (1,680 s at 5 Hz for each comparison). **a** Composite of experimental segment data. **b** Composite of 8, individually calibrated random walkers from Fig. 10. **c** Single random walker realisation, calibrated with weighted average parameter values found in (14)

Our final observation was a comparison between the trajectories of the eight individually calibrated random walks described above, to the trajectory of a single random walker, parameterised with the weighted-average values given in (14). The plots in Fig. 11a–c) indicate, respectively, the relative density of positions in the tank/simulation area for experimental swimming trajectories, composite density of the eight individually calibrated random walkers, and the trajectory density of a single random walker simulated with the parameter set defined in (14), computed for an equivalent duration ($28 \times 60=1{,}680\,\hbox {s})$. Trajectories in Fig. 11a indicate a clear preference of zebrafish to swim in close proximity to the tank walls with minimal departures into the centre of the tank. In comparison, the density plot for the individually calibrated simulations (Fig. 11b) indicated increased variation in the area explored by random walkers, with more activity in the central region. By performing a weighted average across all available swimming data (Fig. 11c), specifying a set of appropriate mean-model parameters, we found that a single (1,680 s duration) realisation of the model reproduced a comparable density structure to that of the composite individuals, with significant wall-following behaviour and only a slightly increased frequency of departures toward the centre.

## Conclusions

A model of spontaneous zebrafish motion has been presented which captures the approximate distribution of speed and angular speed of swimming fish, accounting for both the autocorrelation and interdependence of these processes. Analysis of simulated trajectories suggests that our model describes many of the salient features of zebrafish locomotion, including the emergence of a thigmotactic-like (wall-following) behaviour when model parameters are calibrated on fish exhibiting similar patterns of motion. Specifically we find that this ‘passive’ wall-following behaviour results from a model in which only repulsion from the wall is present. The novel feature of this model, extending the ‘Persistent Turning Walker’ model due to Gautrais et al., is to capture the intrinsic speed variation of zebrafish and other small fish.

Importantly, by allowing speed to vary in our model, further progress can be made in the development of group models which can address the most recent experimental findings for similar fish species. We refer specifically to the findings of Katz et al. (2011) and Herbert-Read and Perna (2011), which report that speed regulation is the primary response governing the interaction between conspecifics and their environment.

Further development of these models, informed directly from experimental data, represents a significant departure from some canonical approaches where fish are modelled with constant speed and conspecific interactions result in changes only to their heading direction, or angular speed. Direct calibration of the model to experimentally observed fish trajectories results in a purely data-driven model and provides the necessary foundations for the future objective of understanding modelling the dynamics of multi-fish shoals. The results of the model are encouraging and provide a solid basis for future investigations into fish social response.

## Electronic supplementary material

Below is the link to the electronic supplementary material.

ESM 1 (EPS 251 kb)

ESM 2 (TIFF 689 kb)

ESM 3 (GFC 247 kb)

ESM 4 (EPS 345 kb)

ESM 5 (EPS 423 kb)

ESM 6 (EPS 327 kb)

ESM 7 (EPS 958 kb)

ESM 8 (GFC 558 kb)

ESM 9 (EPS 246 kb)

ESM 10 (TIFF 1,297 kb)

ESM 11 (TIFF 888 kb)

ESM 12 (EPS 777 kb)

ESM 13 (EPS 458 kb)

ESM 14 (EPS 1,025 kb)

ESM 15 (PDF 84 kb)

## Footnotes

1 Random walk trajectories with a duration of 60 s are computed in approximately 0.2 s in the current implementation with $\varDelta t = 0.2\,\hbox {s}$

2 We denote ‘passive’ thigmotactic-like behaviour as occurrences of wall-following which is not driven by explicit modelling of psychological effects, for example in seeking protections from predators
