# Supplementary figures and images for: Data-driven stochastic modelling of zebrafish locomotion
Source: J Math Biol. 2014 Oct 31;71(5):1081–105. doi: 10.1007/s00285-014-0843-2 (PMC4598355; doi:10.1007/s00285-014-0843-2)

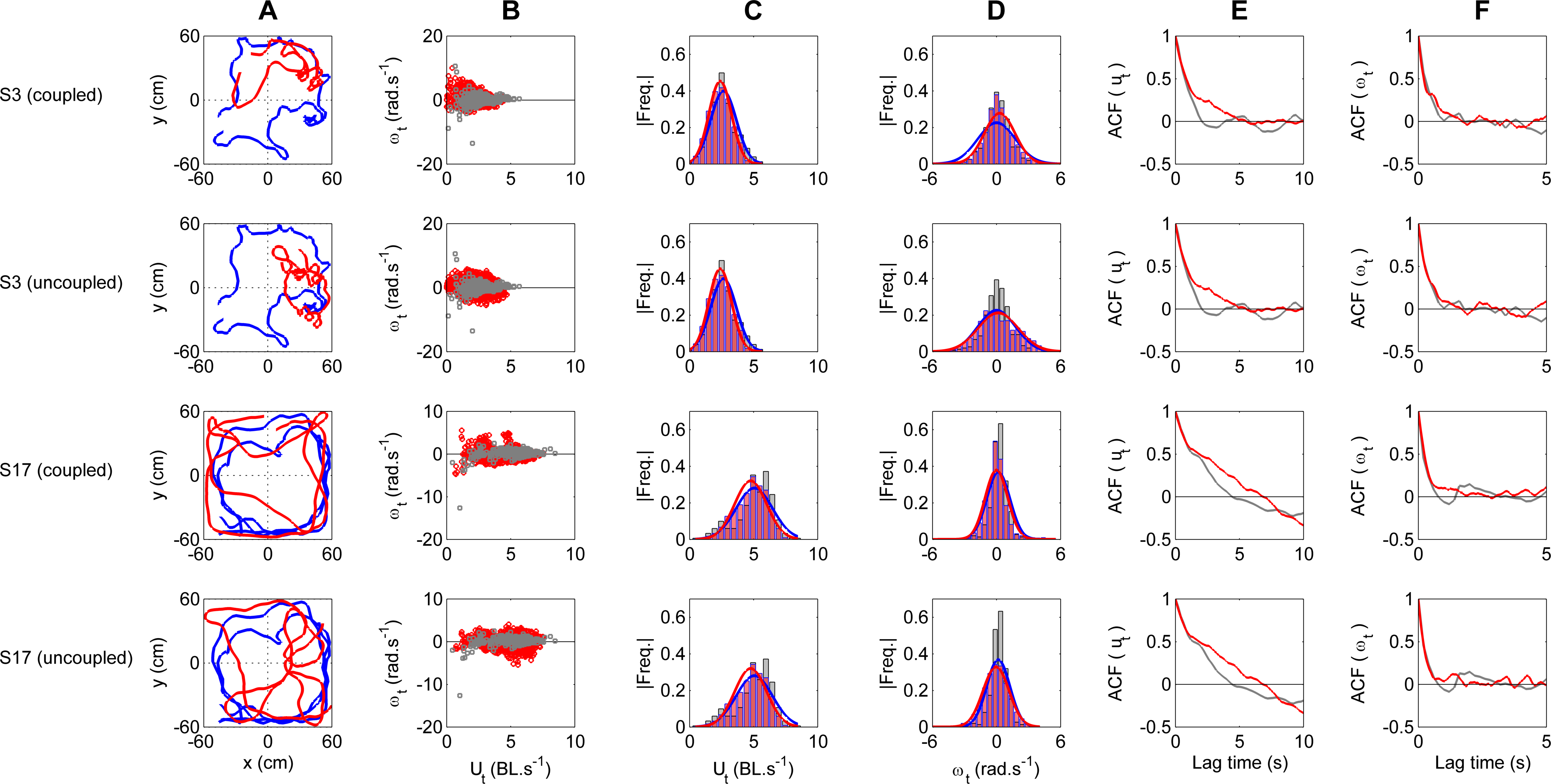

Supplement: Supplementary file 2 — ESM 2 (TIFF 689 kb) [file 285_2014_843_MOESM2_ESM.tiff]

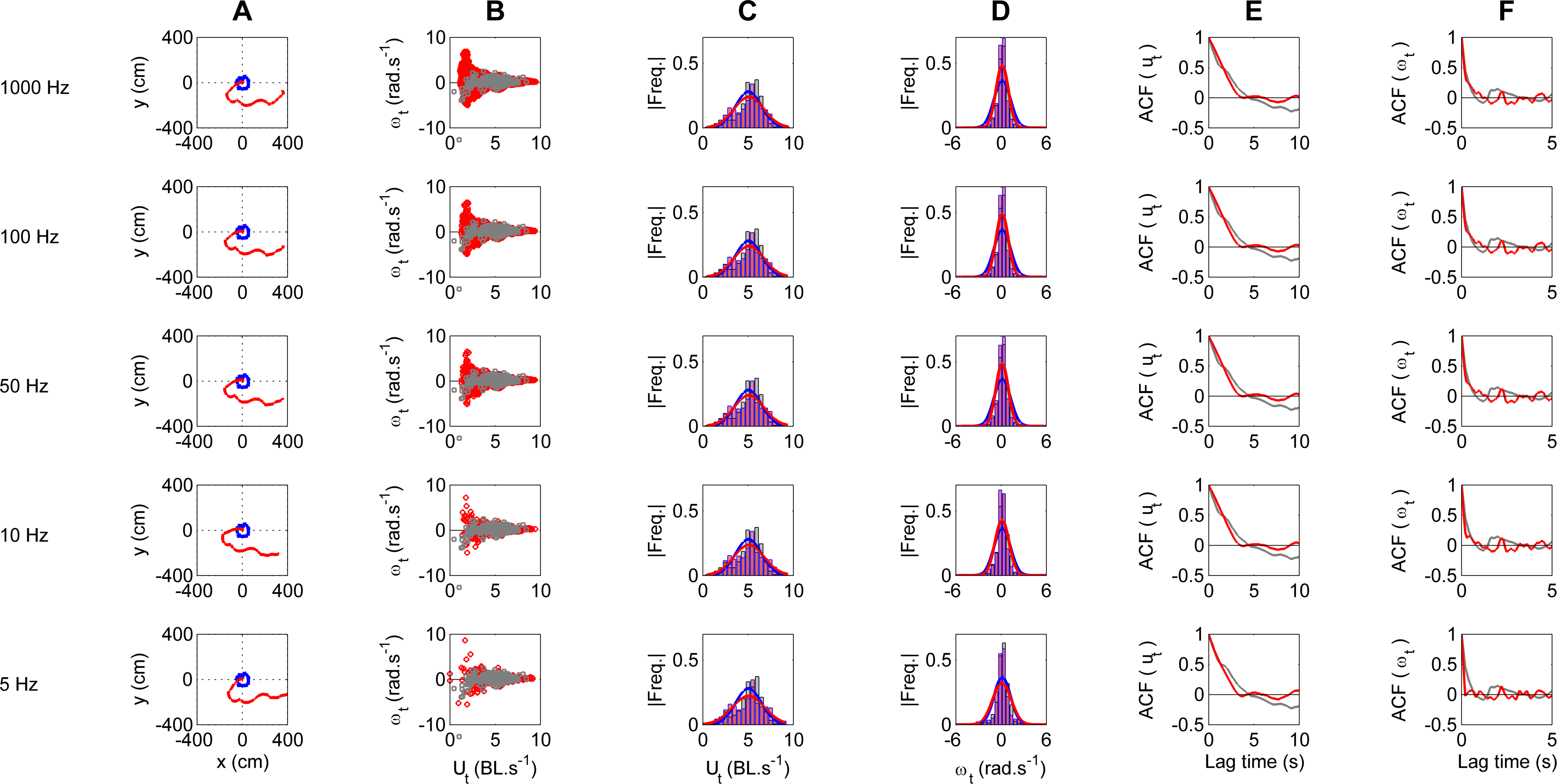

Supplement: Supplementary file 8 — ESM 8 (GFC 558 kb) [file 285_2014_843_MOESM8_ESM.tiff]

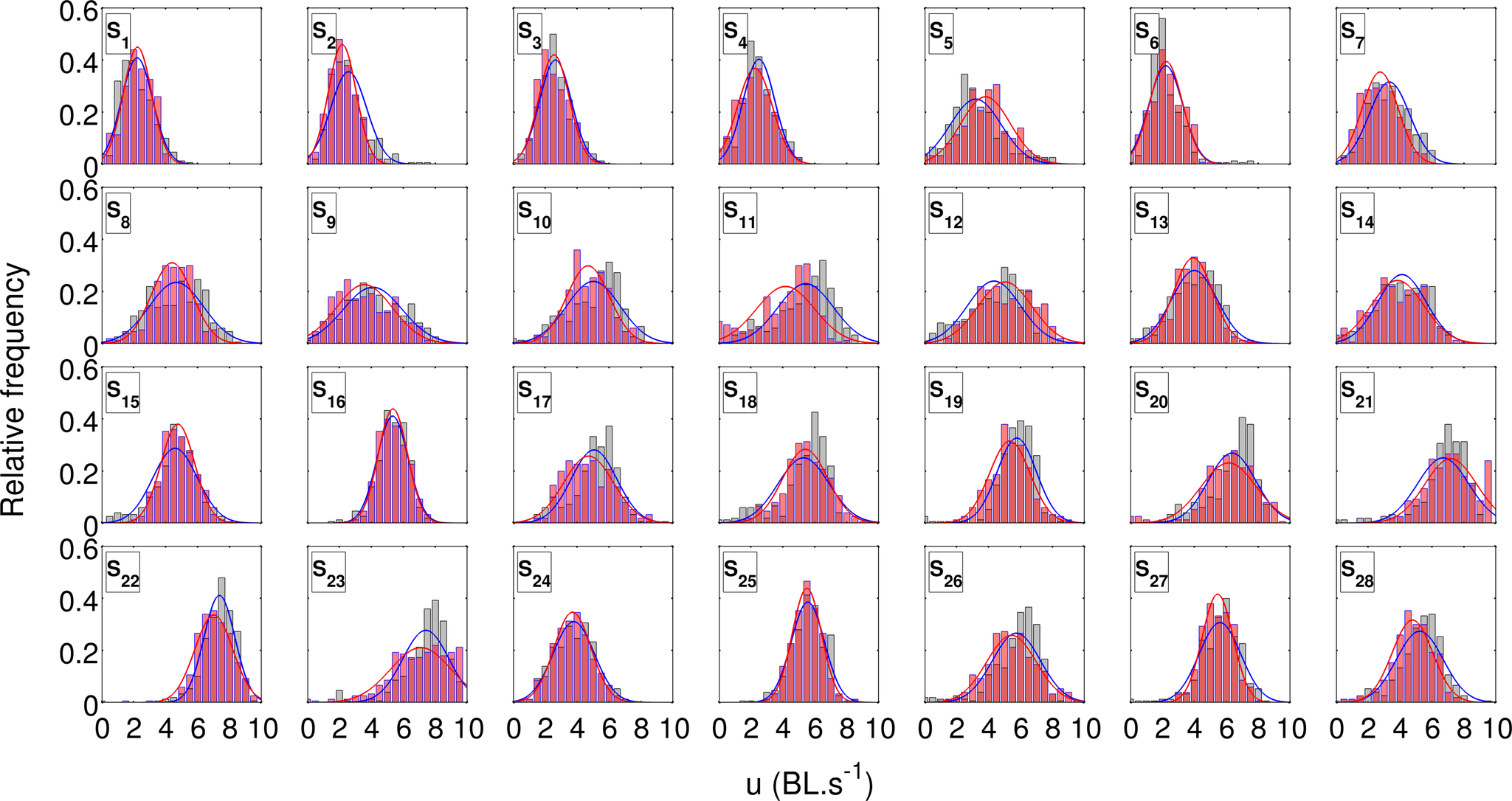

Supplement: Supplementary file 10 — ESM 10 (TIFF 1,297 kb) [file 285_2014_843_MOESM10_ESM.tiff]

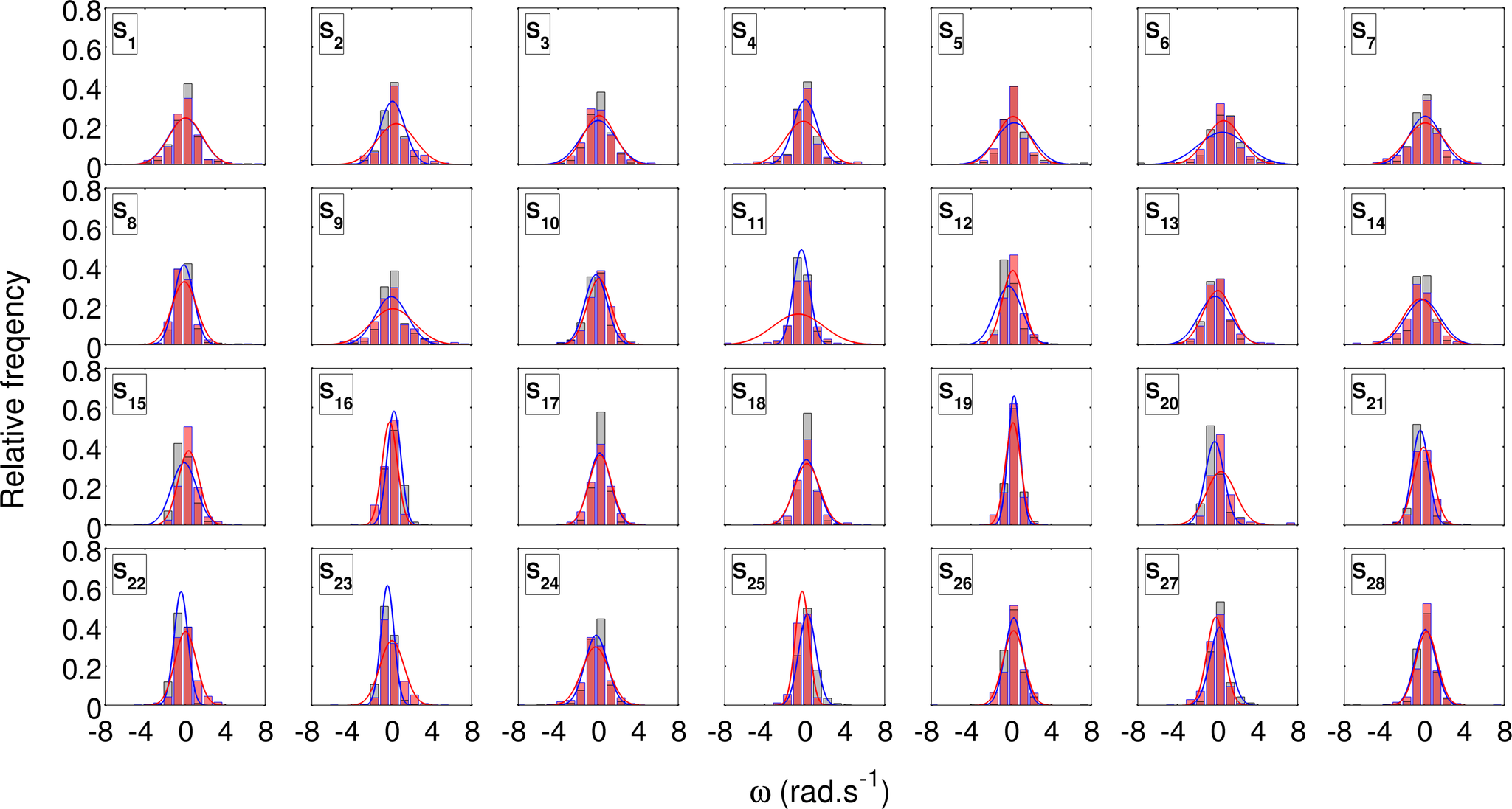

Supplement: Supplementary file 11 — ESM 11 (TIFF 888 kb) [file 285_2014_843_MOESM11_ESM.tiff]
