# Supplementary material for: Data-driven stochastic modelling of zebrafish locomotion
Source: J Math Biol. 2014 Oct 31;71(5):1081–105. doi: 10.1007/s00285-014-0843-2 (PMC4598355; doi:10.1007/s00285-014-0843-2)
Supplement: Supplementary file 15 — ESM 15 (PDF 84 kb) [file 285_2014_843_MOESM15_ESM.pdf]

Supplementary information

Fig. S1 Swimming filter – effect of pre-processing on speed. Time series plots of the instantaneous speed u_t of raw observation data for 10 zebrafish, with 28 isolated segments each representing 1 min of active swimming (green) as determined by the pre-processing filter. Periods in which the speed of an individual drops below a threshold value of 1 BL.s^{-1} (3 cm.s^{-1}) for longer than 2 s were rejected. From the remainder of the data, 60 s periods of sustained swimming were isolated, with segments denoted $S_1 \ldots S_{28}$. Segments of data less than 60 s in duration are discarded (e.g. remaining F_6 data after segment S_{19} is 58.2 s in duration and is therefore discarded)

Fig. S2 Swimming filter: effect of pre-processing on turning speed. Time series plots of the turning speed ω_t of raw observation data for 10 zebrafish, with 28 isolated segments each representing 1 min of active swimming (green) as determined by the pre-processing filter. Erratic motion, or thrashing, was found to coincide with periods of near-stationary (forward) motion and thus was largely rejected by the filter, as required.

Fig. S3 Wall-corrected turning speed as a function of projected collision distance. Computed values of the wall-corrected turning speed ω_c are plotted against projected collision distance d_W for all experimental data segments (*grey squares*), and compared with data from individually calibrated random walkers (*red circles*). Non-parametric regression (thin dashed) and exponential fits (thick solid) are shown for experimental (*red*) and simulated (*blue*) data. The effect of the wall avoidance function $f_w(d_W, \phi_W)$ implemented in our model, is to bend RW trajectories away from the boundaries in a broadly similar manner to that observed experimentally. Where there was sufficient data to interpolate the exponential functions for both experimental and simulated data, a similar functional dependence on d_W was found for some segments. However, due to the intrinsic stochasticity of the trajectory data, these plots are intended primarily to show the general trend for simulated trajectories to exhibit a similar skewed distribution of ω_c as a function of d_W.

Fig. S4 Wall-corrected turning speed as a function of projected collision time. Computed values of the wall-corrected turning speed ω_c are plotted against projected collision time t_W for all experimental data segments (*grey squares*), and compared with data from individually calibrated random walkers (*red circles*). Non-parametric regression (thin dashed) and exponential fits (thick solid) are shown for experimental (*blue*) and simulated (*red*) data. Similar to the data shown in Fig. S3, we found a broadly similar (skewed) distribution of ω_c as a function of t_W due to wall interactions governed by the wall-avoidance function $f_w(d_W, \phi_W)$ (dependant on the projected collision *distance* d_W).

Fig. S5 Fish speed as a function of projected collision distance. Values of speed u_t are plotted against projected collision distance d_W for all experimental data segments (*grey squares*), and compared with data from individually calibrated random walkers (*red circles*). Non-parametric regression (dashed lines) are shown for experimental (*blue*) and simulated (*red*) data. Finding no strong trend in the distribution of speed u_t as a function of projected wall collisions, we do not include an explicit speed dependence in our model. In these plots we find that both experimental and simulated speed data exhibit similarly flat responses as a function of d_W.

Fig. S6 Fish speed as a function of projected collision time. Values of speed u_t are plotted against projected collision time t_W for all experimental data segments (*grey squares*), and compared with data from individually calibrated random walkers (*red circles*). Non-parametric regression (dashed lines) are shown for experimental (*blue*) and simulated (*red*) data. Finding no strong trend in the distribution of speed u_t as a function of projected wall collisions, we do not include an explicit speed dependence in our model. In these plots we find that both experimental and simulated speed data exhibit similar flat responses as a function of t_W.

Fig. S7 Effects of simulation timestep / sample frequency on trajectory data. To study the robustness of simulated trajectories with respect to the numerical timestep value Δt and corresponding sample generation frequency f_s (Hz) - we compare unbounded RW trajectories, simulated using identical stochastic processes (dW_t and dZ_t) sampled at different frequencies, with model parameters calibrated for experimental segment S_17. Columns A-F show respectively: 60 s RW trajectory portraits; speed-turning speed correlation; speed distribution, turning speed distribution; speed and turning speed autocorrelation. Each row represents trials computed with sample frequencies ranging from 1000 Hz to 5 Hz (Δt =0.001 s – 0.2 s). Experimental segment data (grey) is compared to the RW realisations (red) for each trial.

Fig. S8 Comparison between experimental segment trajectories and corresponding random walker. Trajectory portraits for individually calibrated random walkers *(thick blue lines)*, simulated for 60 s, compared with experimental source trajectories *(thin lines coloured according to parent fish ID)*. Qualitative comparisons are gleaned from these trajectory portraits which indicate differing patterns of swimming behaviour across each of the segments. Some trajectories followed more tightly curving, or erratic paths, whilst others appeared to follow more fluid, open curves. The unique parameter calibration of the SDEs driving each random walker was found in many examples to reflect these different characteristics, producing similar curvature to the corresponding source data. For example, the tightly winding trajectory of segment S_6 is well captured by the model, producing a similarly curving random walker path. In contrast, the trajectory of S_{24} is found to be more fluid, punctuated by occasional rapid turns and exhibiting strong thigmotactic-like behaviour, where both features are again reflected in the corresponding random walker trajectory.

Fig. S9 Comparison of speed distribution. Speed U_t distributions of independently calibrated random walkers, simulated for 60 s at 5 Hz (*red*), compared with the associated experimental segment distribution (*grey*). Normal pdf for simulated data and experimental data are indicated by *red* and *blue* curves respectively. Simulated speed data was found, as expected, to be normally distributed with means and variances closely matched to that of the source data segments.

Fig. S10 Comparison of turning speed distribution. Turning speed Ω_t distributions of independently calibrated random walkers, simulated for 60 s at 5 Hz (*red*), compared with the associated experimental segment distribution (*grey*). Normal pdf for simulated data and experimental data are indicated by *red* and *blue* curves respectively. Turning speed data was consistently more peaked than would be expected for a normal distribution, due to the coupling between SDEs, and therefore compared more favourably with experimental data across all segments.

Fig. S11 Comparisons for speed autocorrelation function — ACF$_u$. Speed ACFs for independently calibrated random walkers (*red*) are compared with corresponding ACFs from experimental data (*grey*). The autocorrelation functions for both speed and turning speed (Fig. S12) for all segments are well matched between experimental and simulated realisations. Subject to intrinsic noise, and the effects of the boundaries on turning, these ACF comparisons indicate that the main features of the autocorrelation, primarily the decay rates, remain consistent with those of the corresponding experimental processes u_t and ω_t. However, by employing the MLE calibration method described by van den Berg, 2011, we find that estimation of rate parameter θ is dominated by the lag-one autocorrelation coefficient r_1 as we described in (6). Recovering the initial ACF decay accurately is therefore sensitive to the data acquisition rate f_s and the smoothness of the experimental ACF, under the assumption that it decays exponentially.

Fig. S12 Comparisons for turning speed autocorrelation function - ACF$_\omega$. Turning speed ACFs for independently calibrated random walkers (*red*) are compared with corresponding ACFs from experimental data (*grey*). The autocorrelation functions for both speed (Fig. S11) and turning speed for all segments are well matched between experimental and simulated realisations. Subject to intrinsic noise, and the effects of the boundaries on turning, these ACF comparisons indicate that the main features of the autocorrelation, primarily the decay rates, remain consistent with those of the corresponding experimental processes u_t and ω_t. However, by employing the MLE calibration method described by van den Berg, 2011, we find that estimation of rate parameter θ is dominated by the lag-one autocorrelation coefficient r_1 as we described in (6). Recovering the initial ACF decay accurately is therefore sensitive to the data acquisition rate f_s and the smoothness of the experimental ACF, under the assumption that it decays exponentially. The oscillations observed in the experimental ACF$_\omega$ functions are found to be more pronounced in trajectories which exhibit stronger thigmotactic behaviour, reflecting the periodic quarter turns at the tank corners, with a period dependant on the swimming speed. Similar oscillations, for example those in ACF$_\omega$ for S_{23} and other simulated segments exhibiting strong wall-following, are observed with a comparable period.

Fig. S13 Comparing cross-correlation of U_t and Ω_t for simulated and experimental segments. The cross-correlation between speed and turning speed of independently calibrated random walkers are compared with corresponding experimental data. The joint distributions of speed and turning speed values for each segment confirm that the coupling function f_c enforces a suitable cross-correlation between U_t and Ω_t, uniquely parameterised by MLE calibrated values of μ_u and σ_ω for each segment ($\sigma_0 = 10\,\text{rad.s}^{-1}$ fixed). For each simulated segment comparison, we recovered the characteristic tapering of the range and variance of Ω_t values which decrease at higher speeds.

Fig. S14 Effects of coupling function f_c on trajectory data. To study the effects of the additional coupling between speed and turning speed - decribed by f_c, two representative segments S_3 and S_{17} were used to calibrate random walkers, simulated with and without the coupling function (fixed σ_ω equal to calibrated segment value). Columns A-F show respectively: 60 s RW trajectory portraits; speed-turning speed correlation; speed distribution, turning speed distribution; speed and turning speed autocorrelation. Rows contain data from each trial, with experimental segment data (grey) compared to the RW realisations (red).
